# Nose‐to‐Brain Delivery of Acyl‐Ghrelin Peptide Gold Nanoconjugates for Treatment of Neurodegenerative Diseases

**DOI:** 10.1002/smll.202504517

**Published:** 2025-07-20

**Authors:** Shunping Han, Rifka N. Utami, Yue Qin, Revadee Liam‐Or, Jemeen Sreedharan, Jeffrey S. Davies, Khuloud T. Al‐Jamal

**Affiliations:** ^1^ Institute of Pharmaceutical Science Faculty of Life Sciences & Medicine King's College London Franklin‐Wilkins Building 150 Stamford Street London SE1 9NH UK; ^2^ Department of Basic and Clinical Neuroscience The Maurice Wohl Clinical Neuroscience Institute Institute of Psychiatry Psychology and Neuroscience King's College London London SE5 9RT UK; ^3^ Molecular Neurobiology Institute of Life Sciences School of Medicine Swansea University Swansea SA2 8PP UK

**Keywords:** acyl‐ghrelin peptide, brain uptake, gold nanorods, intranasal administration, neurodegenerative diseases

## Abstract

Neurodegenerative diseases remain a major therapeutic challenge in aging populations. Acyl‐ghrelin, a 28‐amino acid gut hormone, demonstrates neuroprotective effects but is limited by instability, rapid clearance, and non‐specific distribution when systemically delivered. Nose‐to‐brain delivery using nanotechnology offers a promising alternative. Gold nanorods (AuNRs), with high therapeutics loading capacity, are proposed as carriers for intranasal acyl‐ghrelin delivery. The previous study demonstrates that intranasal AuNRs effectively reach the brain with minimal systemic exposure. This work investigates the feasibility of using acyl‐ghrelin gold nanoconjugates to deliver and retain its pharmacological activity through intranasal administration for neurodegenerative diseases. Acyl‐ghrelin is conjugated via its C‐terminus to hetero‐functional polyethylene glycol (PEG) using EDC/sulfo‐NHS coupling chemistry, then attached to AuNRs through stable Au─S bonds. Reaction conditions are optimized to minimize multi‐PEG substitution, preserving acyl‐ghrelin bioactivity and preventing AuNR cross‐linking. The resulting nanoconjugates successfully deliver ghrelin to the brain, reaching peak levels at 10 min post‐administration with ≈2067.6 ± 760.6 pg g^−1^ of brain, a fourfold increase over native expression. Importantly, the peptide retains biological function, as evidenced by AMPK phosphorylation at 30–60 min, a key marker of ghrelin‐induced neuroprotection. These findings support intranasal AuNR‐mediated delivery of acyl‐ghrelin as a promising strategy for treating neurodegenerative diseases.

## Graphical abstract

Intranasal delivery of acyl‐ghrelin using gold nanorods (AuNRs) is investigated as a strategy for treating neurodegenerative diseases. Acyl‐ghrelin is conjugated to AuNRs under optimized conditions that preserve its bioactivity and prevent nanoparticle cross‐linking. The resulting nanoconjugates efficiently reach the brain and activate neuroprotective pathways, highlighting their potential for peptide‐based central nervous system therapeutics.

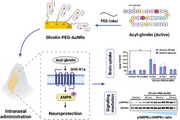

## 1 Introduction

Neurodegenerative diseases including Alzheimer's disease (AD), Parkinson's disease (PD), and amyotrophic lateral sclerosis (ALS) represent a significant unmet medical need in our aging society, for which effective treatments are lacking.^[^ ^1^ ^]^ Peptides, proteins, and antibodies are of increasing interest in biomedical fields due to their high potency and selectivity. Peptides biodegrade into nontoxic metabolites, possess a minimal potential for drug‐drug interactions, and are less likely to cause an immunogenic reaction compared to larger proteins.^[^ ^2^, ^3^ ^]^ However, their widespread application for the treatment of neurodegenerative diseases is limited due to insufficient brain accumulation, poor chemical and physical stability impacted by enzymatic degradation, and rapid elimination from the circulation.^[^ ^2^ ^]^ Despite peptide drugs accounting for a significant proportion of the pharmaceutical market, there are no peptide drugs available for the treatment of neurodegenerative diseases.^[^ ^2^, ^3^ ^]^

Ghrelin, a 28‐amino acid gut‐hormone, demonstrates therapeutic benefits in neurodegenerative diseases, including reducing neuroinflammation, modulating microglial activity, and protecting against neuron death in rodent models with AD, PD, and ALS.^[^ ^4^, ^5^, ^6^ ^]^ Depending on the presence or absence of the acyl group at the third serine residue, ghrelin can be divided into acyl‐ghrelin and des‐acyl ghrelin.^[^ ^7^ ^]^ Only acyl‐ghrelin, the active form, can bind to its cognate receptor (GHS‐R1a), activate signaling, and exert neuroprotective effects.^[^ ^8^ ^]^ Our previous work identified that intraperitoneally delivered acyl‐ghrelin suppresses toxic microglial activation^[^ ^5^ ^]^ and promotes hippocampal neurogenesis in the adult mammalian brain^[^ ^9^ ^]^ and that this ghrelin‐induced neuroprotection requires activation of AMPK in dopamine neurons.^[^ ^5^ ^]^ Conversely, des‐acyl ghrelin reduced hippocampal neurogenesis and plasticity in mice.^[^ ^10^ ^]^ Although it has been reported that ghrelin can penetrate the blood‐brain barrier (BBB),^[^ ^11^ ^]^ the acyl‐ghrelin undergoes deacylation to des‐acyl ghrelin by serum and tissue esterases such as butyrylcholinesterase (BuChE).^[^ ^12^ ^]^ Its short plasma half‐life of 9–13 min^[^ ^13^ ^]^ and non‐specific distribution make systemic administration of acyl‐ghrelin inadequate as a therapeutic molecule.

Nanotechnology is a strategy to improve the utilization of peptide drugs. Peptide‐nanoparticle complexes offer advantages over free peptides in enhancing stability, prolonging retention time in the biological environment, altering biodistribution profiles, and increasing their bioavailability at the site of action.^[^ ^14^, ^15^ ^]^ Gold nanoparticles (AuNPs) are outstanding materials in nanomedicine due to their optical and electronic properties^[^ ^16^ ^]^ and have been considered one of the most stable and least toxic metal nanomaterials.^[^ ^15^ ^]^ Surface functionalization of AuNPs with peptides is possible through non‐covalent and covalent bonds such as electrostatic interactions and formation of the strong gold‐thiol (Au─S) bonds, respectively.^[^ ^17^ ^]^ In the last decade, a variety of peptide‐AuNPs conjugates have been synthesized with peptides acting as therapeutic agents and cellular targeting moieties.^[^ ^18^ ^]^ AuNPs have been proven to protect the conjugated peptides and preserve their functions against enzyme degradation, primarily owing to the steric hindrance provided by the AuNPs core.^[^ ^19^, ^20^ ^]^ Therapeutic peptides including insulin, anti‐cancer peptides, and pro‐apoptotic peptides demonstrate enhanced efficacy and improved pharmacodynamic profiles when delivered as peptide‐AuNPs conjugates compared to the soluble peptide.^[^ ^20^, ^21^, ^22^, ^23^ ^]^ AuNPs are easy to fabricate in different sizes, shapes, and structures. The morphology of AuNPs affects the efficacy of the conjugates.^[^ ^18^ ^]^ Compared to spherical gold nanoparticles (AuNSs) of a similar diameter, gold nanorods (AuNRs) offer advantages such as near‐infrared imaging capability, benefits in photo‐thermal therapy and imaging, and favorable biological behavior due to their elongated morphology. For example, studies demonstrate that AuNRs prolong circulation times, reduce liver accumulation, and have higher tumor accumulation compared to their spherical counterparts.^[^ ^24^, ^25^ ^]^ Also, for comparable brain levels of PEGylated AuNSs and AuNRs, AuNSs show higher levels in the kidney, spleen, liver, and lung after intraperitoneal injection.^[^ ^26^ ^]^

In our previous study, we confirmed the potential of AuNRs as nose‐to‐brain carriers to treat brain diseases.^[^ ^27^ ^]^ AuNRs enter the brain in a rapid, effective, and non‐invasive manner with minimal systemic exposure following intranasal administration. In this study, acyl‐ghrelin was conjugated via its C‐terminus to hetero‐functional polyethylene glycol (PEG) using EDC/sulfo‐NHS coupling chemistry. Acyl‐ghrelin bearing PEG was conjugated to AuNRs via the stable Au─S bond. Reaction conditions were optimized to minimize the formation of di‐ and tri‐(PEG) substituted acyl‐ghrelin, which can reduce the bioactivity of acyl‐ghrelin, which if it occurs can result in cross‐linking of AuNRs. Brain uptake and in vivo bioactivity of acyl‐ghrelin gold nanoconjugates via the nose‐to‐brain route were then investigated. To the best of our knowledge, this is the first study to demonstrate the feasibility of using intranasally administered acyl‐ghrelin gold nanoconjugates for brain targeting, with quantitative assessment of both brain concentration and bioactivity. Our findings provide a new approach for the treatment of neurodegenerative diseases.

## 2 Results

### 2.1 Reaction Optimization to Favor Mono (PEG)‐Substituted Acyl‐Ghrelin Synthesis

**Scheme 1:**
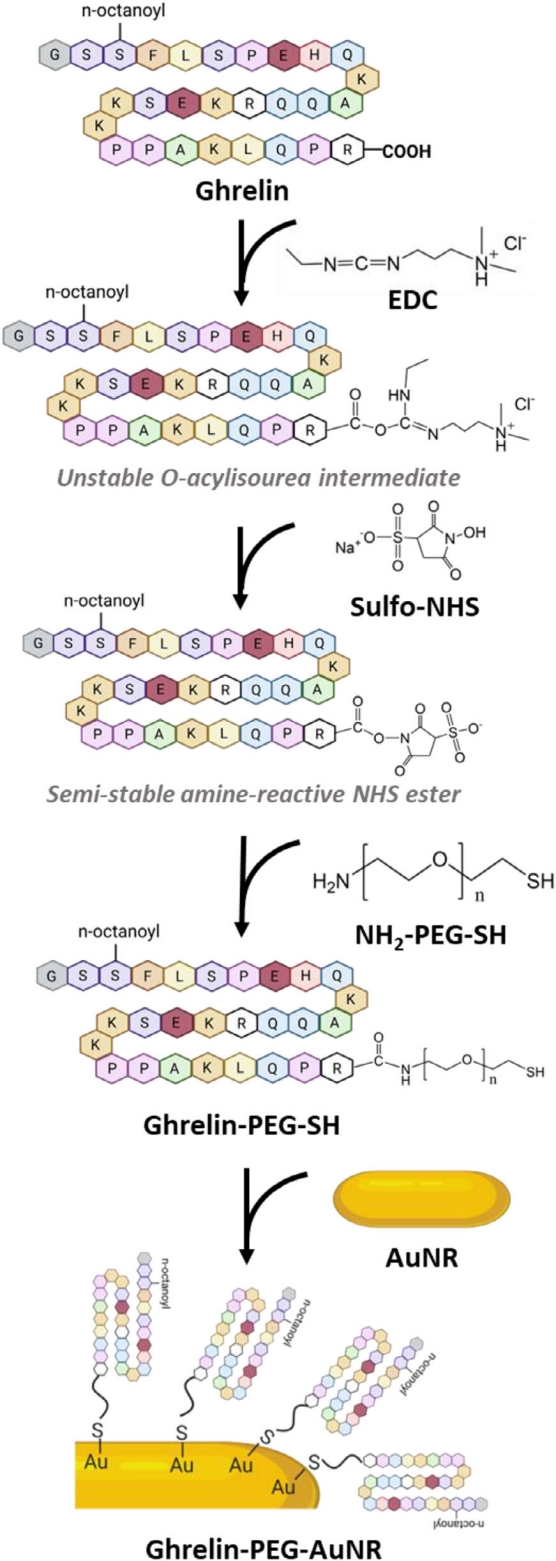
Schematic illustration representing ghrelin‐PEG‐AuNR conjugation. The carboxyl groups in the C‐terminus or glutamic acid residues at positions 8 and 17 in acyl‐ghrelin are the potential targeted sites for NH_2_‐PEG‐SH conjugation. Reaction conditions were optimized to favor the formation of mono‐PEG substituted ghrelin at its C‐terminus.

Structure‐activity analysis of acyl‐ghrelin illustrated that the N‐terminal tetrapeptide of acyl‐ghrelin is crucial for its binding to GHS‐R receptor while the C‐terminus of the peptide, ghrelin (16′−28′), had no affinity to the receptor.^[^ ^28^, ^29^ ^]^ In this study, we therefore aimed to activate the carboxyl group in the C‐terminus of acyl‐ghrelin using EDC/sulfo‐NHS coupling chemistry to be coupled to the amino group in NH_2_‐PEG‐SH as described in **Scheme** **1**.

In a linear polypeptide containing multiple carboxyl groups, the carboxyl group at the C‐terminus is typically involved in peptide bond formation during chemical coupling reactions. In acyl‐ghrelin, in addition to the C‐terminus, glutamic acid amino acid residues at positions 8 and 17, each possessing a free carboxyl group, are also potential conjugation sites for PEGylation. To increase PEGylation efficiency of the peptide while avoiding formation of di‐ and tri‐substituted acyl‐ghrelin resulting from PEGylation of glutamic acid residues, the molar ratio of ghrelin and NH_2_‐PEG‐SH was optimized to favor mono (PEG)‐substituted ghrelin synthesis.

**Figure 1:**
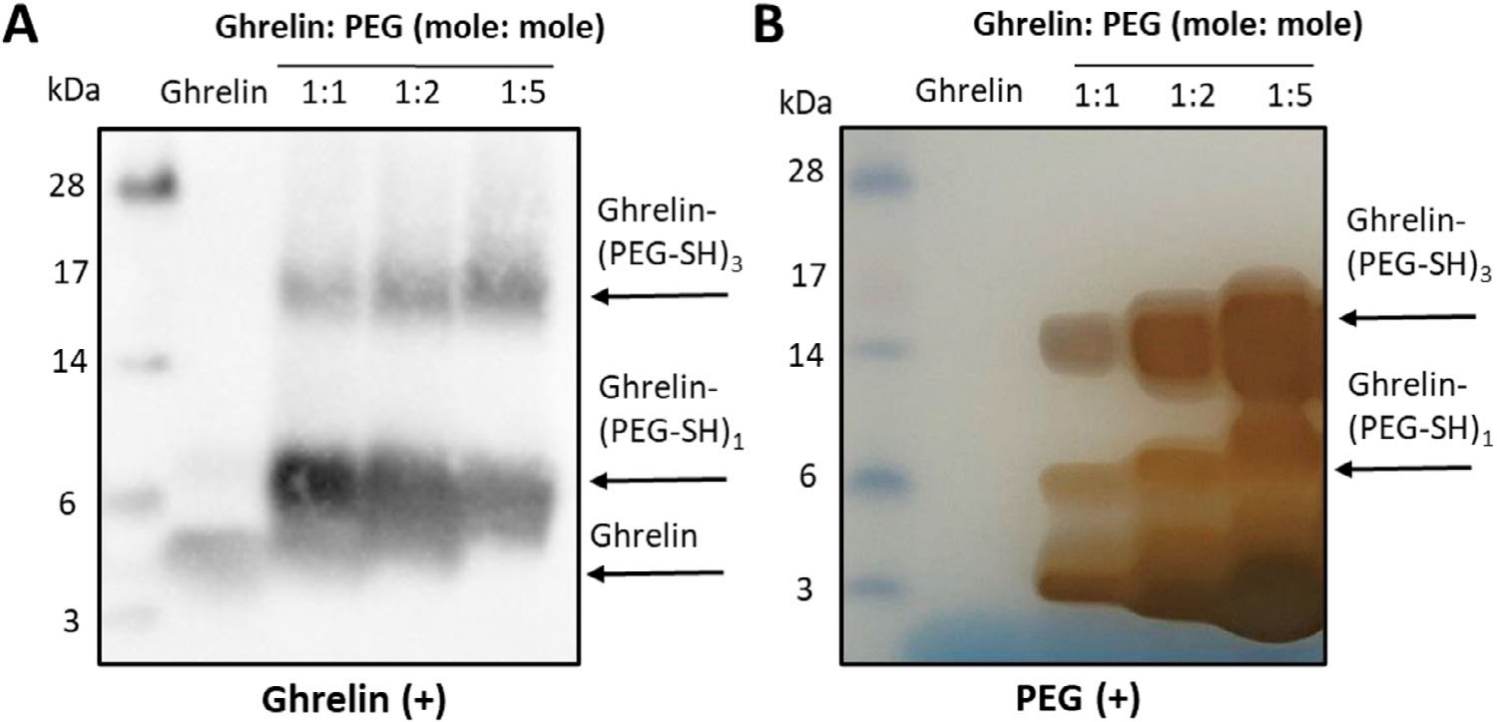
Optimization of ghrelin‐PEG‐SH synthesis reaction conditions. A) SDS‐PAGE analysis of the reaction mixture containing 10 µg ghrelin by Western blotting analysis and B) iodine staining confirmed that a ghrelin‐to‐NH_2_‐PEG‐SH molar ratio of 1:1 resulted in the highest yield of monofunctionalized ghrelin‐PEG, (ghrelin‐(PEG‐SH)_1_).

As shown in **Figure** **1**, an equimolar ratio of NH_2_‐PEG‐SH and ghrelin favored formation of mono (PEG)‐substituted ghrelin, ghrelin‐(PEG‐SH)_1_ (MW ≈7 kDa) confirmed by Western blotting (for ghrelin visualization, Figure 1A) and iodine staining (for PEG visualization, Figure 1B).^[^ ^30^ ^]^ Increasing PEG excess to 2‐fold and 5‐fold resulted in stronger bands of tri‐(PEG)‐substituted acyl‐ghrelin or ghrelin‐(PEG‐SH)_3_ (MW ≈15 kDa) and reduced intensity of ghrelin‐(PEG‐SH)_1_ bands. It was concluded that the synthesis condition using 40‐fold molar excess of sulfo‐NHS to activate the carboxyl groups of ghrelin (Figure S1, Supporting Information), followed by conjugation to NH_2_‐PEG‐SH at a 1:1 molar ratio, resulted in optimal conditions for synthesizing mono (PEG)‐substituted ghrelin. Removal of unconjugated PEG and ghrelin from the conjugate was achieved by gel chromatography using a NAP™‐5 column prior to conjugation with AuNRs (Figure S2, Supporting Information).

### 2.2 Preparation and Physicochemical Characterization of Ghrelin‐PEG‐AuNRs

Ghrelin‐PEG‐SH and NH_2_‐PEG‐SH (as non‐drug control) were conjugated to AuNRs via an Au─S bond. Ghrelin loading determined using the Ghrelin (Active) ELISA Kit was 386 ± 192 mole ghrelin/mole AuNRs. The physicochemical characterization of AuNRs, PEG‐AuNRs, and ghrelin‐PEG‐AuNRs are summarized in **Table** **1** and Table S1 (Supporting Information).

**Table 1:** Physicochemical properties of ghrelin‐PEG‐AuNRs.

Compound	Ghrelin to AuNR graft ratio^b)^	Size^c)^	Zeta potential^d)^
	(mole:mole)	(nm)	(mV)
AuNRs	–	46.1 ± 2.5	55.0 ± 4.9
PEG‐AuNRs^e)^	–	79.2 ± 4.7	30.2 ± 0.3
Ghrelin‐PEG‐AuNRs^a)^	386 ± 192	132.2 ± 9.5	18.6 ± 1.5

^a)^ Ghrelin‐PEG‐AuNRs was synthesized at an EDC: sulfo‐NHS: ghrelin: NH_2_‐PEG‐SH molar ratio of 20: 40: 1: 1;

^b)^ Measured by the ghrelin (Active) ELISA Kit;

^c)^ Measured by nanoparticle tracking analysis (NTA);

^d)^ Measured by Zetasizer Nano series;

^e)^ PEG graft ratio to AuNRs is expected to be in the range of 1000–3000 based on our previous experiments.^[^ ^27^ ^]^

**Figure 2:**
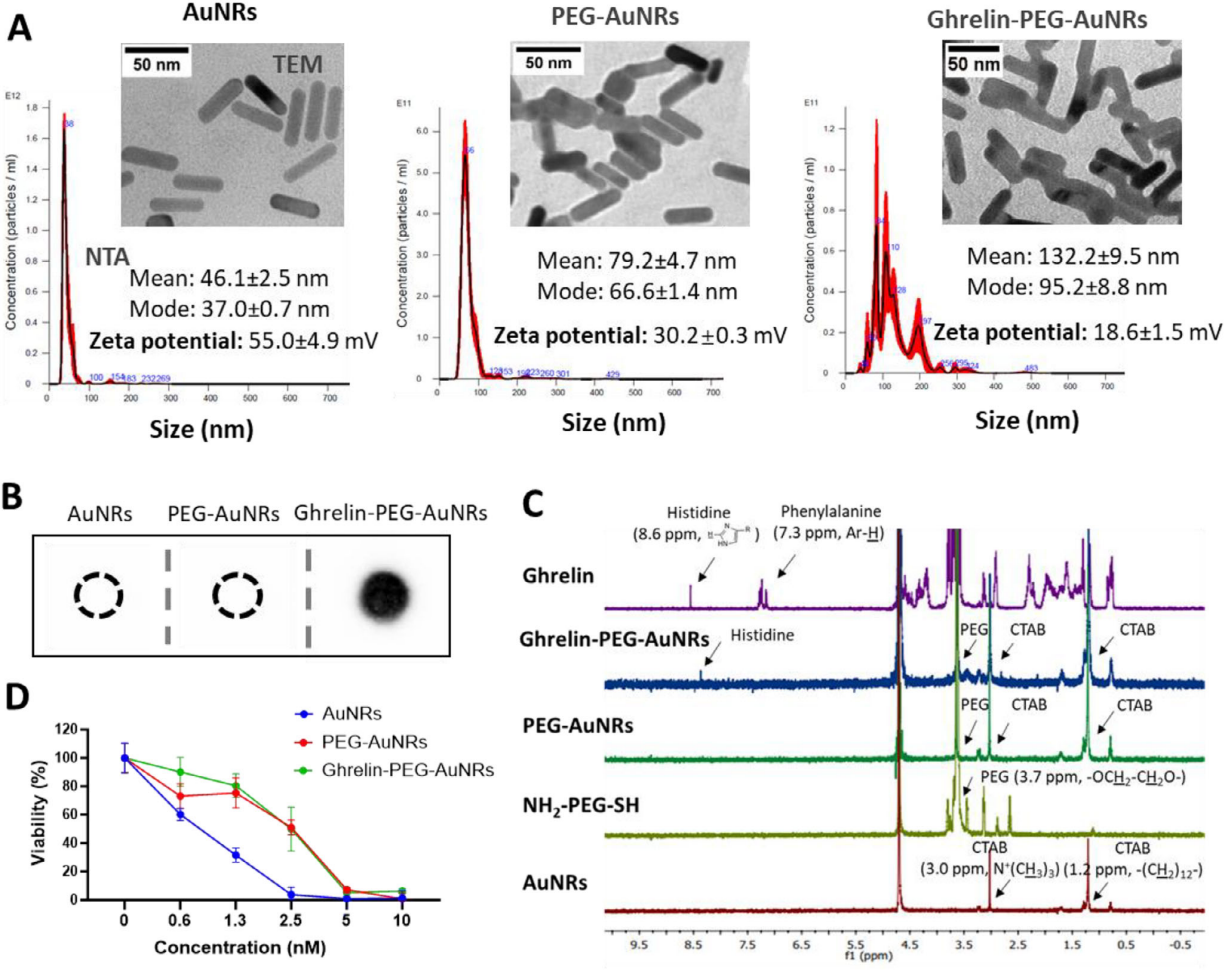
Physicochemical properties and biological evaluation of ghrelin‐PEG‐AuNRs and their precursors. A) Nanoparticle tracking analysis (NTA), transmission electron microscopy (TEM), dynamic light scattering. B) Dot blot immunostaining. C) ^1^H NMR spectra analysis confirming the presence of PEG and ghrelin on AuNRs. D) Cytotoxicity of the AuNRs constructs in SN4741 cells at the 24 h timepoint using the modified LDH assay. Data is expressed as mean ± SD, n = 5. Altogether, ghrelin‐PEG‐AuNRs were successfully synthesized and demonstrated improved biocompatibility compared to as‐synthesized AuNRs.

AuNRs (as‐synthesized) and PEG‐AuNRs (non‐ghrelin PEG functionalized), as controls, demonstrated narrow particle size distribution with the hydrodynamic size and zeta potential of 46.1 ± 2.5, 79.2 ± 4.7 nm and 55.0 ± 4.9, 30.2 ± 0.3 mV, respectively, determined by NTA and Zetasizer Nano series (Table 1 and **Figure** **2**A). After functionalization with ghrelin‐PEG‐SH, the particle size increased to 132.2 ± 9.5 nm with a zeta potential of 18.6 ± 1.5 mV. Representative TEM images demonstrated uniform morphology and high rod purity of the synthesized rods with a length and width of 37.5 ± 4.5 and 10.5 ± 1.3 nm, respectively. In the case of ghrelin‐PEG‐AuNRs, aggregation was observed, possibly caused by cross‐linking induced by ghrelin‐(PEG‐SH)_3_ by‐products. Sizes observed under TEM agreed with hydrodynamic sizes. Only ghrelin‐PEG‐AuNRs stained positive for ghrelin by dot‐blot immunostaining among ghrelin‐PEG‐AuNRs, PEG‐AuNRs, and AuNRs (Figure 2B). Ghrelin without thiol PEG showed negligible affinity to AuNRs by dot blot analysis, confirming that ghrelin binding to AuNRs is covalent (Figure S3, Supporting Information).

Characterization with ^1^H NMR spectra confirmed the presence of PEG and ghrelin/PEG peaks in the PEG‐AuNRs and ghrelin‐PEG‐AuNRs, respectively (Figure 2C). The peaks attributed to CTAB (≈3.0 ppm, N^+^(C *H_3_*)_3_ and ≈1.2 ppm, ‐(C *H_2_*)_12_‐), the surfactant used during synthesis, were observed in all AuNRs spectra, suggesting that CTAB could not be completely eliminated during synthesis.^[^ ^31^ ^]^ The typical sharp peak of PEG (‐OC *H_2_*‐C *H_2_* O‐) repeat units at ≈3.6 ppm was present in both PEG‐AuNRs and ghrelin‐PEG‐AuNRs. In ghrelin‐PEG‐AuNRs, a peak at ≈8.6 ppm corresponding to the imidazole proton of histidine (a residue in ghrelin) was also detected. Interestingly, the phenylalanine signal (≈7.3 ppm), another residue in ghrelin, was not observed. This absence is possibly due to the restricted molecular motion and enhanced relaxation effects caused by phenylalanine's interaction with the hydrophobic CTAB bilayer and potential adsorption onto the gold surface. In contrast, histidine remains more solvated and mobile, allowing its signal to persist.

All conjugates showed dose‐dependent toxicity in a mouse embryonic substantia nigra‐derived cell line, SN4741 cells, used as a GHS‐R expressing model.^[^ ^5^ ^]^ Among them, the as‐synthesized AuNRs, which contained the highest amount of CTAB, were the most toxic (Figure 2D). PEGylation of AuNRs, with and without ghrelin, improved biocompatibility compared to unmodified AuNRs.

The characterization data collectively confirmed the successful loading of ghrelin onto AuNRs, resulting in hydrodynamic sizes of <150 nm. Additionally, ghrelin‐PEG‐AuNRs exhibited improved biocompatibility.

### 2.3 Acyl‐Ghrelin was Successfully Delivered to the Brains via AuNRs Following Intranasal Administration

**Figure 3:**
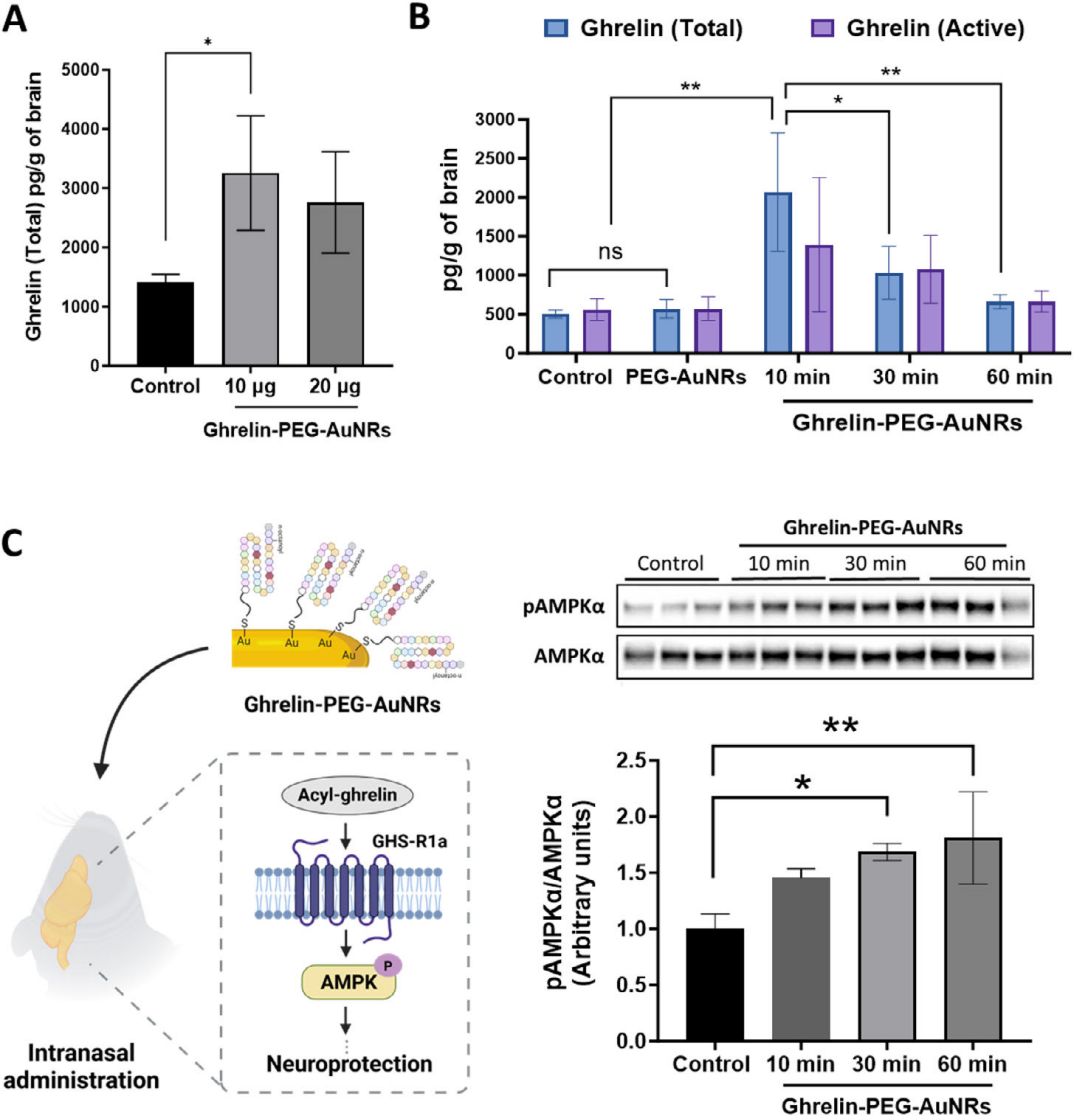
Brain uptake and bioactivity of ghrelin‐PEG‐AuNRs after intranasal administration. C57BL/6 mice were intranasally administered with ghrelin‐PEG‐AuNRs at A) 10 or 20 µg ghrelin equivalent and sacrificed at 10 min post‐administration or B) 10 µg ghrelin equivalent and sacrificed at 10, 30, or 60 min post‐administration. Tissue lysates were analyzed using the total or active ELISA kits for quantification of total and acyl‐ghrelin, respectively. C) Ghrelin‐PEG‐AuNRs induced AMPK phosphorylation at 30 and 60 min post‐administration as evidenced by the representative Western blot images of pAMPKα and AMPKα and quantification of pAMPKα/AMPKα levels in brain lysates. Values were expressed as mean ± SD, n = 3. ^*^ *p* < 0.05, ^**^ *p* < 0.01.

In our in vivo studies, CTS solution (0.5% CMC, 0.1% Tween 20, and 0.9% NaCl, w/w) was used to ensure sufficient retention of the nanoparticles in the nasal passage and was administered to mice as a control. Significantly higher ghrelin levels were detected in brains of mice administered intranasally with 10 µg or 20 µg ghrelin equivalent (*p* > 0.05) per mouse in the form of ghrelin‐PEG‐AuNRs compared with the control mice (*p* < 0.05) at 10 min post‐administration, as measured using Ghrelin (Total) ELISA kit (**Figure** **3**A). A time‐response study demonstrated that the highest ghrelin concentration following intranasal administration was obtained at the 10 min timepoint (≈2067.6 ± 760.6 pg g^−1^ of brain), which decreased at 30 min (1031.7 ± 339.5 pg g^−1^ of brain) and returned to physiological levels at 60 min (660.6 ± 89.0 pg g^−1^ of brain) (Figure 3B). To ensure that ghrelin in the brain was in the active, acyl‐ghrelin form, the ghrelin concentration was measured using the Ghrelin (Active) ELISA kit. Results confirmed that ghrelin detected was in its active form, with no significant difference between total and active ghrelin levels (*p* > 0.05). Additionally, the PEG‐AuNRs (no ghrelin) group showed similar results to the vehicle group, suggesting no interference from AuNRs.

The AMPK signaling pathway is a crucial target for ghrelin‐induced neuroprotection. Our previous work demonstrated that ghrelin‐mediated AMPK phosphorylation, following intraperitoneal administration, was essential for its neuroprotective effects in a mouse model of Parkinson's Disease.^[^ ^5^ ^]^ In this study, ghrelin‐PEG‐AuNRs induced AMPK phosphorylation at 30 and 60 min following intranasal administration, as evidenced by the increased pAMPKα/AMPKα ratio in brain lysates, measured by Western blotting (Figure 3C). The pAMPKα/AMPKα ratio in the PEG‐AuNRs group was similar to that of the control group, suggesting that the AMPK phosphorylation was attributed to the conjugated ghrelin peptides rather than the nanoparticle vehicles (Figure S4, Supporting Information).

## 3 Discussion

Acyl‐ghrelin has demonstrated promising therapeutic potential for central nervous system (CNS) diseases, including AD, PD, Huntington's disease, and ALS.^[^ ^7^, ^32^, ^33^, ^34^ ^]^ For example, ghrelin has been shown to disrupt amyloid beta (Aβ) plaques, inhibit tau protein hyperphosphorylation, and mitigate mitochondrial dysfunction, offering potential benefits for AD treatment.^[^ ^32^ ^]^ Body weight loss, systemic and cellular metabolism impairments, and insulin‐like growth factor‐1 (IGF‐1) reductions are highly associated with faster disease progression and worsening disease outcomes in ALS patients. Acyl‐ghrelin‐mediated pathways can improve body weight regulation, metabolism, and the anabolic and neuroprotective actions of growth hormone and IGF‐1, and have been considered promising therapeutic targets in ALS.^[^ ^34^ ^]^ Acyl‐ghrelin has a short half‐life (≈9–13 min in human plasma) due to rapid degradation by esterases such as BuChE in circulation.^[^ ^12^, ^13^ ^]^ Due to its peptidic nature, it undergoes first pass metabolism in the liver and rapid renal elimination. The cognate receptor of acyl‐ghrelin, GHS‐R1a, is expressed not only in multiple areas of the brain but also in peripheral tissues, including the stomach, adrenal and pituitary gland, pancreas, intestines, and liver.^[^ ^7^, ^35^ ^]^ Adverse effects such as somnolence, a warm feeling, facial warmth, abdominal pain, emesis, and vertigo were observed after systemic administration of ghrelin.^[^ ^36^, ^37^ ^]^ Taken together, parenteral delivery of ghrelin for treatment of CNS diseases is not preferred and may necessitate the use of high doses, resulting in systemic toxicity and undesired side effects.

Intranasal administration is an alternative route for delivering therapeutic agents to the brain. This delivery route is non‐invasive. It allows therapeutics to enter the brain directly through the olfactory and trigeminal nerve pathways, bypassing first‐pass metabolism in the liver and reducing drug accumulation in non‐target tissues.^[^ ^38^ ^]^ Major challenges in nose‐to‐brain delivery of peptides include physical and biological instability of the therapeutics impacted by nasal enzymatic degradation, rapid mucociliary clearance, and limited permeation across the nasal mucosa.^[^ ^39^ ^]^ The application of a suitable delivery carrier that can overcome these challenges will increase the brain bioavailability of therapeutics.

Therapeutic peptides can be incorporated into nanoparticles through either physical encapsulation/absorption or chemical conjugation, favoring nose‐to‐brain delivery.^[^ ^40^, ^41^ ^]^ To date, only liposomes have been proposed as a nanoplatform for intranasal ghrelin delivery. Salade et al. encapsulated ghrelin in chitosan‐coated liposomes designed for nose‐to‐brain delivery.^[^ ^42^ ^]^ This work, conducted as part of a doctoral thesis,^[^ ^43^ ^]^ demonstrated that the liposomes effectively protected ghrelin from enzymatic degradation by trypsin and carboxylesterase. The chitosan coating improved mucin adsorption and enhanced permeation across an in vitro Calu3 epithelial monolayer. A preliminary in vivo investigation was included in the thesis, in which fluorescently labeled ghrelin‐loaded liposomes were intranasally administered to mice for qualitative evaluation of brain delivery. Confocal fluorescence imaging detected weak signals in the olfactory bulb, suggesting successful brain targeting. However, the detected signal was limited and observed only in a small number of fluorescent particles. Peptide‐nanoparticle conjugation is an alternative approach to deliver therapeutic peptides. The sufficiently high packing density of peptides on nanoparticles creates steric hindrance, preventing enzymes from accessing the cleavage sites and causing degradation.^[^ ^20^ ^]^ It potentially reduces the likelihood of undesired burst release, which is often encountered in the encapsulation/absorption loading method, and thus offers prolonged protection of the therapeutics. This protective strategy, achieved through ligand conjugation to nanoparticles, has also been demonstrated in shielding nucleic acids from nuclease degradation.^[^ ^44^ ^]^

In this study, AuNRs were judged suitable as a delivery system for ghrelin based on several key advantages over conventional platforms such as polymeric or lipid‐based nanoparticles. Their large surface area and well‐defined surface chemistry allow for efficient and site‐specific conjugation of peptides or other therapeutic agents via strong Au─S bond formation, enabling a high loading capacity. AuNRs also demonstrate high colloidal and structural stability under physiological conditions, reducing the risk of premature drug release or degradation. Furthermore, AuNRs are generally regarded as having low toxicity and favorable biocompatibility. Emerging studies have highlighted therapeutic properties associated with AuNRs, including anti‐inflammatory and antioxidant effects, which support neuronal repair and reduce neuroinflammation.^[^ ^45^ ^]^ Additionally, we have previously demonstrated the feasibility of nose‐to‐brain delivery of AuNRs and established its brain distribution, which occurs within minutes followed by clearance within an hour.^[^ ^27^ ^]^ In this study, AuNRs were administered in a high‐viscosity, isotonic aqueous dispersion containing 0.5% CMC, 0.1% Tween 20, and 0.9% NaCl (w/w), which was hypothesized to slow down the mucociliary clearance to prolong exposure to the olfactory mucosa, thereby further enhancing ghrelin's delivery to the brain.

Understanding the structure‐activity relationship of acyl‐ghrelin is crucial in designing its nanoconjugates. It has been widely established that *n*‐octanoylation of the serine residue, the third amino acid from the N‐terminus, is essential for ghrelin binding to GHS‐R receptor.^[^ ^7^, ^8^ ^]^ A study by Matsumoto et al. using several rat ghrelin fragments demonstrated that the minimum core required for ghrelin activity resides in the N‐terminal tetrapeptide while the C‐terminus of the peptide, ghrelin (16′−28′), had no affinity to the ghrelin receptor.^[^ ^28^ ^]^ In this study, we therefore chose to conjugate ghrelin via its C‐terminus to the amine terminus of PEG using EDC/sulfo‐NHS coupling chemistry. Although we acknowledge that the glutamic acid residues at positions 8 and 17 in ghrelin are also potential conjugation sites, it is not expected that this conjugation will diminish ghrelin activity. PEG was used as a spacer to maintain the flexibility of ghrelin for its receptor recognition and to improve both colloidal and enzymatic stability and biocompatibility of AuNRs.^[^ ^46^ ^]^ Despite optimizing the reaction conditions to favor the synthesis of ghrelin‐(PEG‐SH)_1_, the presence of unconjugated ghrelin and ghrelin‐(PEG‐SH)_3_ could not be eliminated. We have shown that unconjugated ghrelin exhibited minimal adsorption to AuNRs due to the absence of a thiol group in its structure. Ghrelin‐(PEG‐SH)_3_, however, may have caused cross‐linking of AuNRs, leading to the apparent increase in hydrodynamic size following conjugation to ghrelin‐PEG‐SH and potentially lowering the effective ghrelin grafting ratio. Critically, NTA measurements performed following each batch of ghrelin‐PEG functionalization consistently confirmed that the majority of particles were ≈100 nm in size. Particles larger than 200 nm accounted for less than 10% of the population, and particles in the micrometer range were rarely detected (Figure 2A; Table S1, Supporting Information). Given this minimal proportion of aggregates, we do not expect a substantial impact on the overall biological performance. Nevertheless, to achieve more precise control over conjugation, minimize even this minor cross‐linking, and enhance the ghrelin to AuNR graft ratio, we propose to orthogonally functionalize acyl‐ghrelin with PEG during its synthesis on a resin support using solid phase peptide synthesis. Alternatively, an immune affinity beads binding assay using anti‐ghrelin antibodies can be employed to characterize the resulting ghrelin‐PEG‐AuNRs and selectively remove unconjugated AuNRs.

Ghrelin‐PEG‐AuNRs demonstrated similar brain uptake profiles to that of AuNRs which we reported previously to peak at 10 min post‐administration.^[^ ^27^ ^]^ The olfactory nerve and trigeminal nerve pathways are mostly responsible for direct nose‐to‐brain drug delivery. Intranasally delivered therapeutics can reach the olfactory bulbs and other areas of the CNS within several minutes to 30 min via extracellular mechanisms between cells in the olfactory epithelium. Additionally, this nasal barrier could be considered “leaky” to the CNS due to the constant turnover of the olfactory cells which regenerate every 3–4 weeks as a result of exposure to external toxins.^[^ ^47^ ^]^ The alternative route which may take several hours to days is the intracellular route along the olfactory nerve. The brain localization patterns of ghrelin‐PEG‐AuNRs and AuNRs in our studies suggest the extracellular route in the olfactory pathway is the predominant mode of ghrelin transport into the brain. The study by Liu et al. demonstrated comparable t_max_ (1 h) in the brain of coumarin‐6 intranasally delivered by PEG‐PCL nanoparticles with or without lactoferrin modification, but lactoferrin modified PEG‐PCL nanoparticles had higher brain area under the curve (AUC).^[^ ^48^ ^]^ Li et al. reported that intranasal administration of galantamine hydrobromide (GH) loaded liposomes demonstrated 3.3‐fold improvement in AUC but reduced T_max_ from 1.5 to 0.75 h compared to orally administered GH aqueous solution.^[^ ^49^ ^]^ These studies tracked the cargos rather than the nanoparticles. PEG‐PLA nanoparticles also demonstrated rapid brain uptake with the signal being observed at 5 min, peaking at 30 min, and decreasing from 2 h following intranasal administration.^[^ ^50^ ^]^ The brain uptake rate of AuNRs^[^ ^27^ ^]^ and acyl‐ghrelin in this study peaked at ≈10 min post‐administration, which is faster than what has been reported in previous studies. This anomaly remains unexplained. AuNRs‐enabled nose‐to‐brain delivery may therefore be beneficial for CNS therapeutics that require a rapid onset of action.

Doses of acyl‐ghrelin administered to achieve a pharmacological effect in the CNS vary in the published literature.^[^ ^51^ ^]^ Intraperitoneal injection of 80 µg kg^−1^ ghrelin daily for 8 consecutive days was sufficient to reverse memory impairment in ghrelin knockout mice.^[^ ^52^ ^]^ In a study that utilized soluble ghrelin intranasal administration, a dose of 0.04 µg kg^−1^ given at 1 and 24 h after hypoxia‐ischemia induction significantly reduced the percentage of infarcted brain area and improved both short‐term neurobehavioral deficits and long‐term neurological function in a rat model of neonatal hypoxic‐ischemic encephalopathy.^[^ ^53^ ^]^ In this study, we administered a dose of ≈500 µg kg^−1^, which resulted in brain levels of ≈2068 pg g^−1^ of brain. This bioavailability was sufficient to induce AMPK phosphorylation at 30–60 min post‐administration, suggesting that brain concentrations obtained in our study are therapeutically meaningful. Our study is the first to quantitatively demonstrate that intranasal delivery of ghrelin enhances its brain bioavailability, providing direct evidence that nanoparticulate delivery enables rapid brain targeting, thus sparing acyl‐ghrelin from enzymatic degradation in the nasal cavity. These data provide the necessary knowledge to perform follow‐on studies characterizing the putative neuroprotective action of ghrelin‐PEG‐AuNRs in appropriate experimental models of neurodegenerative disease, such as an amyotrophic lateral sclerosis–frontotemporal dementia (ALS‐FTD) mouse model and Parkinson's disease models, including genetic (e.g., alpha‐synuclein) and toxin‐based (e.g., 1‐methyl‐4‐phenyl‐1,2,3,6‐tetrahydropyridine (MPTP)) models.

To support clinical translation, future studies should incorporate comprehensive biodistribution and toxicological assessments. This work builds upon our previous study,^[^ ^27^ ^]^ in which we thoroughly characterized the kinetic biodistribution and regional brain localization of PEGylated AuNRs following intranasal administration. In the current study, ghrelin was conjugated to AuNRs via a stable Au─S bond. Given the structural similarity and identical administration route, we anticipate that ghrelin‐PEG‐AuNRs exhibit a biodistribution pattern comparable to that of PEGylated AuNRs. Indeed, both formulations showed peak brain accumulation ≈10 min post‐administration, supporting this assumption. However, we recognize that conjugation with ghrelin could lead to subtle changes in biodistribution kinetics. Radiolabeling of ghrelin, as well as immunofluorescence (IF) or immunohistochemistry (IHC) staining for ghrelin in brain tissue, should be conducted in future studies to gain a more detailed understanding of the in vivo behavior and brain localization of ghrelin‐PEG‐AuNRs.

Additionally, although gold nanoparticles are considered biologically inert, recent evidence suggests that their safety profile may vary depending on physicochemical properties such as size and surface chemistry. For instance, Lee et al. reported that 5 nm AuNPs induced significantly higher expression of nestin, a marker associated with CNS injury, in immunoreactive cells following stereotactic injection into the cerebral cortex, compared to 100 nm AuNPs, indicating increased neurotoxicity associated with smaller particles.^[^ ^54^ ^]^ Therefore, for intranasally delivered ghrelin‐PEG‐AuNRs, further investigations are essential to assess not only biodistribution and long‐term safety but also the effects of chronic dosing, immunogenicity, and potential off‐target responses. These investigations will be critical for advancing this platform toward clinical application.

## 4 Conclusion

In this study, we reported an optimized functionalization method for the chemical preparation of acyl‐ghrelin peptide AuNR conjugates, which we demonstrate to be successfully delivered to the brain after nasal administration. Acyl‐ghrelin was detected in the brain in its active form and induced AMPK phosphorylation, which is believed to be important for neuroprotection. This proof‐of‐concept study offers a new perspective in enabling acyl‐ghrelin‐based therapeutics for the treatment of neurodegenerative diseases.

## 5 Experimental Section

### Materials

Gold(III) chloride trihydrate (HAuCl_4_ · 3H_2_O), cetyltrimethylammonium bromide (CTAB), sodium borohydride (NaBH_4_), L‐ascorbic acid (Vitamin C), sodium carboxymethyl cellulose (CMC, Mw = ≈ 700,000 Da), iodine, Tween 20, N‐(3‐Dimethylaminopropyl)‐N′‐ethylcarbodiimide hydrochloride (EDC), N‐Hydroxysulfosuccinimide sodium salt (sulfo‐NHS), universal antibody dilution buffer and Pefabloc SC were purchased from Sigma–Aldrich (UK). Silver nitrate (AgNO_3_) was from VWR International (UK). NH_2_‐PEG‐SH (amine‐PEG_3500_‐thiol, average Mw = 3500 Da) was purchased from JenKem Technology (USA). Isoflurane (IsoFlo) for anesthesia was purchased from Zoetis (UK). Dulbecco's modified eagle medium (DMEM, high‐glucose), fetal calf serum (FCS), penicillin‐streptomycin, GlutaMAX™, 0.5% trypsin‐EDTA, SeeBlue™ Plus2 Pre‐stained protein standard, PageRuler™ Plus Prestained Protein Ladder (10 to 250 kDa), NuPAGE™ 4 to 12% Bis‐Tris mini protein gels, NuPAGE™ LDS sample buffer (4X), NuPAGE™ MOPS SDS running buffer (20X), and NuPAGE™ MES SDS running buffer (20X) were purchased from Invitrogen (UK). CytoTox 96 non‐radioactive cytotoxicity assay kit for the modified lactate dehydrogenase (LDH) assay was purchased from Promega (USA). Nitrocellulose membrane (0.22 µm) was purchased from Bio‐Rad (UK). Acyl‐ghrelin (Rat) peptide (hereinafter referred to as ghrelin) was purchased from GL Biochem (China). Ghrelin polyclonal antibody (bs‐0467R‐BSS) was purchased from Stratech (UK). Phospho‐AMPKα (Thr172) (#2535), AMPKα (D63G4) (#5832), and HRP‐linked anti‐rabbit IgG (#7074S) were purchased from Cell Signaling (UK). Tris‐Buffered Saline (TBS) buffer (10x) was purchased from Severn Biotech (UK). Coomassie Brilliant Blue R 250 was purchased from Serva (UK). SuperSignal™ West Femto maximum sensitivity substrate, Triton™ X‐100, and dialysis bags (SnakeSkin™ Dialysis Tubing, MWCO = 3500 Da) were purchased from Thermo Fisher Scientific (UK). NAP™‐5 columns were purchased from GE Healthcare Life Sciences (UK). Rat/Mouse Ghrelin (Total) ELISA kits and Rat/Mouse Ghrelin (Active) ELISA kits were purchased from Merck Life Science (UK). UA‐Zero EM stain and carbon‐coated 300‐mesh copper grids were purchased from Agar Scientific (UK). Other chemicals, not specified here, were all analytical grade chemicals purchased from standard suppliers and used without further purification.

### Synthesis of AuNRs

Gold nanorods were synthesized by the seed‐mediated growth method as described previously.^[^ ^27^ ^]^ First, CTAB solution (5 mL, 0.2 M) was mixed with HAuCl_4_ · 3H_2_O (5 mL, 0.0005 M). Ice‐cold NaBH_4_ (600 µL, 0.01 M) was added to the above mixture, which rapidly turned into a brownish‐yellow color. The solution was vigorously stirred for 2 min and kept in a water bath at 28 °C for 2 h and was used as the seed solution.

For growth solution preparation, HAuCl_4_ · 3H_2_O (45 mL, 0.001 M) was mixed with AgNO_3_ (3.6 mL, 0.004 M) and CTAB (45 mL, 0.1 M). After mixing the solution a few times by inversion, vitamin C (0.72 mL, 0.1 M) and HCl (1.44 mL, 1 M) were added to the solution. Finally, the seed solution (360 µL) was added to the prepared growth solution. The whole mixture was kept at 28 °C overnight to obtain AuNRs.

After synthesis, the mixture was purified by a combinational purification method in which the mixture was first dialyzed (dialysis bag, MWCO = 3500 Da) against deionized water (1500 mL) overnight with the external dialysis buffer exchanged once. The solution was then split into two halves, transferred into two 50 mL centrifuge tubes, and centrifuged (Eppendorf, Centrifuge 5810 R, UK) at 10,000 rpm for 15 min at room temperature (RT). The pellets were resuspended into deionized water (30 mL) and washed one more time before collection.

### Synthesis of Ghrelin‐PEG‐SH

Ghrelin was dissolved in PBS buffer (pH 5.8) to obtain a concentration of 300 µm. The molar ratio between EDC and sulfo‐NHS to ghrelin was optimized to activate the carboxyl groups in ghrelin before being reacted with amine groups in PEG. In brief, a mixture (90 µL) containing EDC and sulfo‐NHS at a fixed molar ratio of 1:2 (EDC: sulfo‐NHS) in PBS 5.8 was added to the above ghrelin stock (300 µL) to achieve sulfo‐NHS to ghrelin molar ratios of 2:1, 4:1, 10:1, 15:1, 20: 1 or 40:1. After activation for 15 min at RT, NH_2_‐PEG‐SH (90 µL, 1 mm in 0.1 M sodium bicarbonate (NaHCO₃) buffer, pH 8.2) was added dropwise to the above mixture and reacted for 4 h at RT. After reaction, samples with an equivalent ghrelin amount of ≈15 µg were loaded onto SDS‐PAGE followed by Coomassie blue staining to visualize ghrelin and confirm its conjugation to PEG (Supporting Information). Images were taken using the Bio‐Rad GelDoc System (UK).

To further optimize the ghrelin to NH_2_‐PEG‐SH molar ratio, EDC:sulfo‐NHS:ghrelin at 20:40:1 molar ratio was reacted with NH_2_‐PEG‐SH at increasing PEG to ghrelin molar ratios. Ninety microliters of solution containing EDC and sulfo‐NHS at 20 and 40 mm final concentrations in PBS 5.8 was added to the above ghrelin stock (300 µL, 300 µm). After activation for 15 min at RT, NH_2_‐PEG‐SH (90 µL, 1, 2, or 5 mm in 0.1 m NaHCO₃ buffer, pH 8.2) was added dropwise to the above mixture to achieve ghrelin to NH_2_‐PEG‐SH at 1:1, 1:2, and 1:5 molar ratios. The mixture was reacted for 4 h at RT. Samples with an equivalent ghrelin amount of ≈10 µg were loaded onto SDS‐PAGE followed by Western blot analysis and iodine solution staining to detect the ghrelin and PEG fragments, respectively. Gels were imaged using a digital camera and a Bio‐Rad GelDoc System.

The reaction was applied to a NAP™‐5 Sephadex G25 size exclusion column and eluted with deionized water for buffer exchange. A total of 7 fractions of 200 µL each were collected and qualitatively analyzed for the presence of ghrelin and PEG using dot‐blot immunostaining and iodine solution method (Supporting Information), respectively. Ghrelin or NH_2_‐PEG‐SH samples was applied to NAP™‐5 columns to monitor the elution times of the starting material compared to the conjugates as controls. Ghrelin‐PEG‐SH was collected from fractions 2 to 4. Ghrelin‐PEG‐SH linkers were synthesized freshly each time before use.

### Synthesis of Ghrelin‐PEG‐AuNRs

Ghrelin‐PEG‐SH or NH_2_‐PEG‐SH was conjugated to AuNRs via an Au─S bond. AuNR pellets were collected by centrifugation (10 000 rpm, 15 min, RT) of the stock solution (0.5 mL, 50 nm). Ghrelin‐PEG‐SH (500 µL, ≈150 µm) stock purified by NAP™‐5 column or NH_2_‐PEG‐SH (500 µL, 150 µm) was added to the AuNR pellet fraction (30 µL) and reacted for 24 h at RT, respectively. As a negative control group, ghrelin‐PEG‐SH stock was mixed with deionized water (30 µL) and reacted under the same condition. At the end of the reaction, the mixtures were centrifuged (10 000 rpm, 15 min, RT). The concentration of ghrelin‐PEG‐SH in the supernatant was measured using Rat/Mouse ghrelin (Active) ELISA according to the manufacturer's instructions. The conjugated ghrelin on AuNRs was calculated by quantifying ghrelin in the supernatant of the experimental group subtracted from the negative control group. Ghrelin‐PEG‐AuNRs and PEG‐AuNRs were resuspended into deionized water and washed twice by centrifugation before use.

### Characterization—Nanoparticle Tracking Analysis and Zeta Potential Measurement

Hydrodynamic size and particle concentration of the functionalized AuNRs were determined by nanoparticle tracking analysis (NTA) using NanoSight LM10 (Malvern Instruments, UK). The particles were diluted with filtered deionized water to obtain 20–80 particles in the viewing frame. The modal size and particle count were measured in quadruplicate, with 30 s as the duration for each recording, and analyzed using the NanoSight NTA 3.2 software (Malvern Instruments, UK). Zeta potential of the particles was determined at RT by electrophoretic mobility measurement using Zetasizer Nano series (Malvern Instruments, UK).

### Characterization—Transmission Electron Microscopy

The morphology of the particles was evaluated using a transmission electron microscope (TEM). A drop of particle sample after purification was loaded onto a carbon‐coated 300‐mesh copper grid and allowed to stand for 3 min. Excess fluid was removed by adsorption to a filter paper. The grid was quickly washed with filtered deionized water once and dried in air. For negative staining, the sample placed on the grid was treated with UA‐Zero EM Stain for 2–3 min. Excess fluid was removed using a filter paper, washed twice with filtered deionized water, and dried in air then the grid was imaged at 80 kV with a FEI Tecnai 12 G2 Spirit with an 11 megapixel Olympus SIS side mount Morada camera. The obtained images were analyzed using ImageJ software (USA). More than 200 nanoparticles were counted for the length and width measurement.

### Characterization—Proton Nuclear Magnetic Resonance (^1^H NMR)

Samples were dissolved or dispersed in deuterium oxide (600 µL) and loaded in Wilmad NMR tubes. ^1^H NMR spectra were recorded using a Bruker Ascend 400 MHz NMR spectrometer (Germany). NMR data analysis was performed using MestreNova software (Spain).

### Characterization—Western Blotting and Iodine Solution Staining of SDS‐PAGE Gel

The reaction mixture containing unreacted ghrelin, unreacted PEG, and ghrelin‐PEG‐SH was resolved using SDS‐PAGE electrophoresis. The reaction samples were mixed with LDS Sample Buffer (4X) and heated at 70 °C for 10 min. The samples with the required ghrelin amount were loaded to 4–12%, Bis‐Tris Mini Protein Gels and run at 90–120 V for ≈1.5 h with MES SDS Running Buffer. The molecular weight of the targets was indicated using SeeBlue™ Plus2 Pre‐stained protein standard.

To visualize PEG and ghrelin‐PEG‐SH, the SDS‐PAGE gels were stained using iodine solution according to the published methods with modifications.^[^ ^30^ ^]^ Briefly, the gels were fixed for 30 min and incubated with a mixture containing 8 mL of deionized water, 2 mL of 5% BaCl_2,_ and 1 mL of 0.1 N iodine solution for 5 min. The gel was washed using deionized water until no brownish color was detected in the gel background and imaged by a digital camera.

To visualize ghrelin and ghrelin‐PEG‐SH, the samples in SDS‐PAGE gels were transferred to nitrocellulose (NC) membranes at 100 mA for 1 h on ice. The NC membranes were washed once using TBS‐T buffer (Tris‐buffered saline buffer with 0.1% Tween 20, v/v) for 5 min at RT. The membranes were blocked with universal antibody dilution buffer for 1 h at RT and incubated with primary ghrelin polyclonal antibody (1:1000) overnight at 4 °C. The membranes were washed three times (10 min each) with TBS‐T buffer then incubated with HRP‐conjugated secondary antibody (1:2000) for 1 h at RT in the dark. After three washes with TBST buffer (10 min each), the membranes were incubated with SuperSignal™ West Femto maximum sensitivity substrate according to the manufacturer's instructions and imaged using the Bio‐Rad GelDoc System. Images obtained were analyzed using Image Lab™ software (Bio‐Rad, USA).

### Characterization—Dot Blotting

Ghrelin‐PEG‐SH fractions after NAP™‐5 column (4 µL) or ghrelin‐PEG‐AuNRs (4 µL, 10 nm of particles) after washing were spotted on NC membranes and gently dried under a nitrogen stream. The membranes were blocked with universal antibody dilution buffer, incubated with primary ghrelin polyclonal antibody, HRP‐conjugated secondary antibody, and SuperSignal™ West Femto maximum sensitivity substrate, and imaged using the Bio‐Rad GelDoc System as described above under the Western blotting section.

### Cell Culture

SN4741 cells, a mouse embryonic substantia nigra‐derived cell line, were cultured using DMEM (high glucose) supplemented with 10% heat‐inactivated fetal calf serum, 1% penicillin‐streptomycin, 1% GlutaMAX™, and 0.6% glucose. Cells were maintained at 37 °C under a humidified atmosphere containing 5% CO_2_ and were subcultured using trypsin‐EDTA at ≈80% confluency.

### Cell Viability

Cell viability was evaluated using the modified LDH assay following the published protocol with some modifications.^[^ ^55^, ^56^ ^]^ Cells were seeded in 96‐well plates at a density of 6 × 10^3^ cells well^−1^ overnight before being treated with 100 µL of AuNRs, PEG‐AuNRs, and ghrelin‐PEG‐AuNRs at a particle concentration of 0.6‐10 nm for 24 h (n = 5). The medium was removed, cells were washed with PBS once, and lysed using 100 µL of 1% Triton™ X‐100 PBS solution for 1 h at 37 °C. The plates were centrifuged at 4000 rpm for 1 h at RT to precipitate nanoparticles and cell debris. Thirty microliters of the supernatant, or the lysis solution as the negative control, were transferred into new 96‐well plates, mixed with 30 µL of LDH reagent, and incubated for 20 min in the dark. The reaction was terminated by adding 30 µL of stop solution to each well. Absorbance was measured at 490 nm in an FLUO star OPTIMA plate reader (BMG Labtech, USA). Results were expressed as the percentage cell viability and calculated using the following equation:

(1) $$\begin{array}{ccc} & & \mathrm{Cell}\;\mathrm{viability}\;\left(\%\right)\\ & & =\frac{A490\;\mathrm{nm}\;\mathrm{of}\;\mathrm{treated}\;\mathrm{cells}\;-\;A490\;\mathrm{of}\;\mathrm{negative}\;\mathrm{control}}{A490\;\mathrm{nm}\;\mathrm{of}\;\mathrm{untreated}\;\mathrm{control}\;\mathrm{cells}-\;A490\;\mathrm{of}\;\mathrm{negative}\;\mathrm{control}}\;\\ & & \;\times \;100 \end{array}$$

### Animals

All animal experiments were performed in compliance with the UK Animals (Scientific Procedures) Act 1986 and UK Home Office Code of Practice for the Housing and Care of Animals Used in Scientific Procedures (Home Office 1989). In vivo experimentation was adhered to the project license approved by the King's College London animal welfare and ethical review body (AWERB) and UK Home Office (PP8950634). C57BL/6 mice (18–30 g, 10–14 weeks old) were obtained from Charles River (UK). Male and female C57BL/6 mice were used and evenly distributed to the different groups as per the Medical Research Council (MRC) guidelines in all in vivo experiments with free access to food and water.

### Brain Uptake and Bioactivity of Ghrelin‐PEG‐AuNRs After Intranasal Administration

Mice were intranasally administered under inhalational anesthesia with a total of 20 µL of ghrelin‐PEG‐AuNRs formulations suspended in CTS solution (0.5% CMC, 0.1% Tween 20, and 0.9% NaCl, w/w) by dosing 2 µL to the left and right nostrils alternatively at a minimum 20 s interval. During administration, mice were positioned in the supine position. For dose response experiments, mice were administered with ghrelin‐PEG‐AuNRs at an equivalent ghrelin amount of 10 or 20 µg, and mice were culled at 10 min (n = 3). For the time‐dependency study, mice were administered with ghrelin‐PEG‐AuNRs at an equivalent ghrelin amount of 10 µg, and mice were sacrificed at 10, 30, and 60 min (n = 3). At the desired timepoint, mice were culled by cervical dislocation. Brains were homogenized and lysed by 1% Triton™ X‐100 containing 0.05 M HCl and 1 mg mL^−1^ Pefabloc SC on ice, and centrifuged at 12 000 g for 20 min at 4 °C for three times. The supernatant fractions were stored at −20 °C for subsequent measurements. Total or active ghrelin in the supernatant was measured using Rat/Mouse Ghrelin (Total) ELISA kits and Rat/Mouse Ghrelin (Active) ELISA kits as per the manufacturer's instructions. CTS solution or PEG‐AuNRs were used as dosing controls.

The bioactivity of ghrelin‐PEG‐AuNRs in the brain following intranasal administration was investigated via AMP‐activated protein kinase (AMPK) phosphorylation using Western blot analysis. The total protein amount in brain lysis was measured by BCA assay according to the manufacturer's instructions. Brain samples with equivalent protein amount of 45 µg each were mixed with LDS Sample Buffer (4X), heated at 70 °C for 10 min and then loaded to 4–12% Bis‐Tris Mini Protein Gels using MOPS SDS Running Buffer for electrophoresis. The molecular weight of the proteins was indicated using PageRuler™ Plus Prestained Protein Ladder (10–250 kDa). After gel electrophoresis, the proteins were transferred to NC membranes at 100 mA for 1 h on ice, followed by incubating with Phospho‐AMPKα (1:1000) at 4 °C overnight and HRP‐conjugated secondary antibody (1:2000) at RT for 1 h. After imaging, the Phospho‐AMPKα antibody and HRP‐conjugated secondary antibody in NC membranes were stripped off using stripping buffer according to the manufacturer's instructions. The NC membranes were re‐probed by incubating with AMPKα (1:1000). Other procedures were the same as described before in the Western blotting section. Images were taken and analyzed by the Bio‐Rad GelDoc System and Image Lab™ software, respectively. Total volume (Int) of the bands was used to represent the protein expression.

### Statistical Analysis

Quantitative results were presented as mean ± standard deviation (SD), where “n” denotes the number of repeats. Statistical differences were examined using one‐way ANOVA, except for the total and active ghrelin brain uptake study, which was analyzed using two‐way ANOVA. All analyses were performed using GraphPad Prism 8 software (v 8.2.1). The *p*‐value <0.05 was considered statistically significant.

## Conflict of Interest

The authors declare no conflict of interest.

## Supporting information



Supporting Information

## Data Availability

The data that support the findings of this study are available from the corresponding author upon reasonable request.
