# Supplementary material for: Nose‐to‐Brain Delivery of Acyl‐Ghrelin Peptide Gold Nanoconjugates for Treatment of Neurodegenerative Diseases
Source: Small. 2025 Jul 20;21(36):e04517. doi: 10.1002/smll.202504517 (PMC12423900; doi:10.1002/smll.202504517)
Supplement: Supplementary file 1 — Supporting Information [file SMLL-21-e04517-s001.docx]

**Supplementary Information**

**Nose-to-Brain Delivery of Acyl-Ghrelin Peptide Gold Nanoconjugates for Treatment of Neurodegenerative Diseases**

Shunping Han^1^, Rifka N. Utami^1^, Yue Qin^1^, Revadee Liam-Or^1^, Jemeen Sreedharan^2^, Jeffrey S. Davies^3^ and Khuloud T. Al-Jamal^1^*

1. Institute of Pharmaceutical Science, Faculty of Life Sciences & Medicine, King's College London, Franklin-Wilkins Building, 150 Stamford Street, London SE1 9NH, United Kingdom
2. Department of Basic and Clinical Neuroscience, The Maurice Wohl Clinical Neuroscience Institute, Institute of Psychiatry, Psychology and Neuroscience, King’s College London, London SE5 9RT, United Kingdom
3. Molecular Neurobiology, Institute of Life Sciences, School of Medicine, Swansea University, Swansea SA2 8PP, United Kingdom

*Corresponding author: Khuloud T. Al-Jamal

E-mail address: khuloud.al-jamal@kcl.ac.uk

Keywords**:** Acyl-ghrelin peptide, gold nanorods, intranasal administration, brain uptake, neurodegenerative diseases

**Supplementary Methods**

**Coomassie Blue Staining of SDS-PAGE Gel**

To visualize ghrelin and confirm its conjugation to PEG, the reaction samples were mixed with LDS Sample Buffer (4X) and heated at 70°C for 10 min. The samples with required ghrelin amount were loaded to 4-12%, Bis-Tris Mini Protein Gels and run at 90 V for 30 min followed by 120 V for ~1 h with MES SDS Running Buffer. The gels were fixed for 30 min using a fixing solution containing 60% absolute ethanol, 7.5% acetic acid and 32.5% deionized water (v/v) and stained using Coomassie blue for another 30 min. The gels were washed using destaining solution containing 5% absolute ethanol, 7.5% acetic acid and 87.5% deionized water (v/v) until no blue color can be detected in the gel background. The images were taken using the Bio-Rad GelDoc System.

**PEG Detection by Iodine Solution-Based Assay**

The PEG fragment in ghrelin-PEG-SH after being eluted using NAP™-5 column was detected using iodine solution-based assay by the published method with modifications [1]. In brief, iodine solution was prepared by dissolving 1.27 g iodine in 100 mL of 2% (w/v) potassium iodide. Fractions after being eluted using NAP™-5 column were diluted with deionized water to final concentrations of PEG within the range of 0 - 5 μM. Samples (100 μL) were transferred into 96-well plates and mixed with iodine solution (2 μL). After shaking for 2 min to ensure adequate mixing, the plate was read at 535 nm by a plate reader (BMG Labtech, UK).

**Supplementary Results**

The molar ratio of EDC to sulfo-NHS was fixed at 1:2 as frequently described in the literature. Sulfo-NHS to ghrelin molar ratio was varied while fixing ghrelin to PEG molar ratio at 1:1. The increase in ghrelin’s molecular weight using SDS-PAGE electrophoresis and Coomassie blue staining confirmed the successful PEGylation of the peptide **(Figure S1)**. Unmodified acyl-ghrelin demonstrated a single band at ~4 kDa. At sulfo-NHS to ghrelin molar ratios > 20-fold, a second band at ~7 kDa appeared in addition to the 4 kDa band suggesting that PEGylation reaction was successful but incomplete. EDC: sulfo-NHS: ghrelin molar ratio of 20: 40: 1 was utilized while additional attempts were made to increase PEGylation efficiency of ghrelin.

**
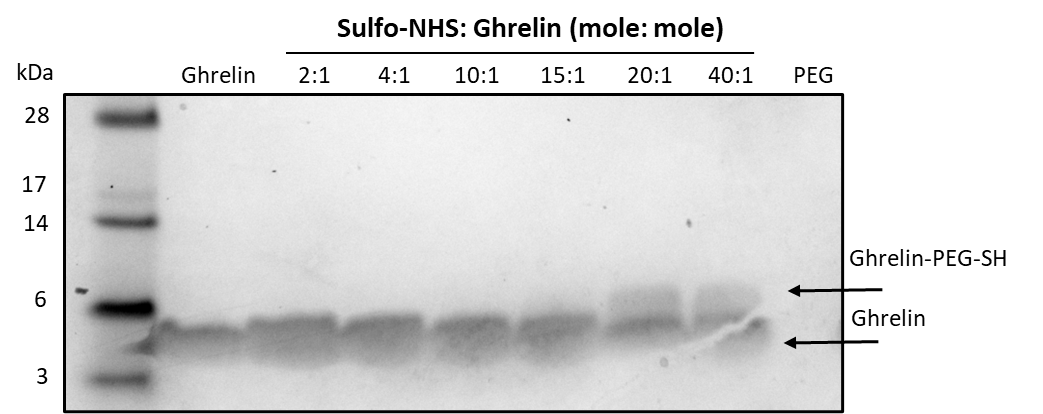
**

**Figure S1**. **Optimization of sulfo-NHS to ghrelin molar ratio.** EDC and sulfo-NHS at fixed 1:2 molar ratio was reacted with ghrelin at increasing molar ratios to activate the ghrelin’s carboxyl groups followed by reaction with the amino group of NH_2_-PEG-SH at ghrelin: NH_2_-PEG-SH molar ratio 1:1. Ghrelin (MW~3.4 kDa, Lane 1) and PEG (MW~3.5 kDa, Lane 8) are loaded as controls. Ghrelin amount per well is equivalent to 15 μg. SDS-PAGE was stained with Coomassie blue and imaged in a Bio-Rad GelDoc System. Increasing the sulfo-NHS molar excess to 20-fold and 40-fold resulted in a second band at ~7 kDa, in addition to the 4 kDa band, suggesting successful conjugation. EDC: sulfo-NHS: ghrelin molar ratio of 20: 40: 1 was used to further optimize ghrelin: NH_2_-PEG-SH molar ratios.

**
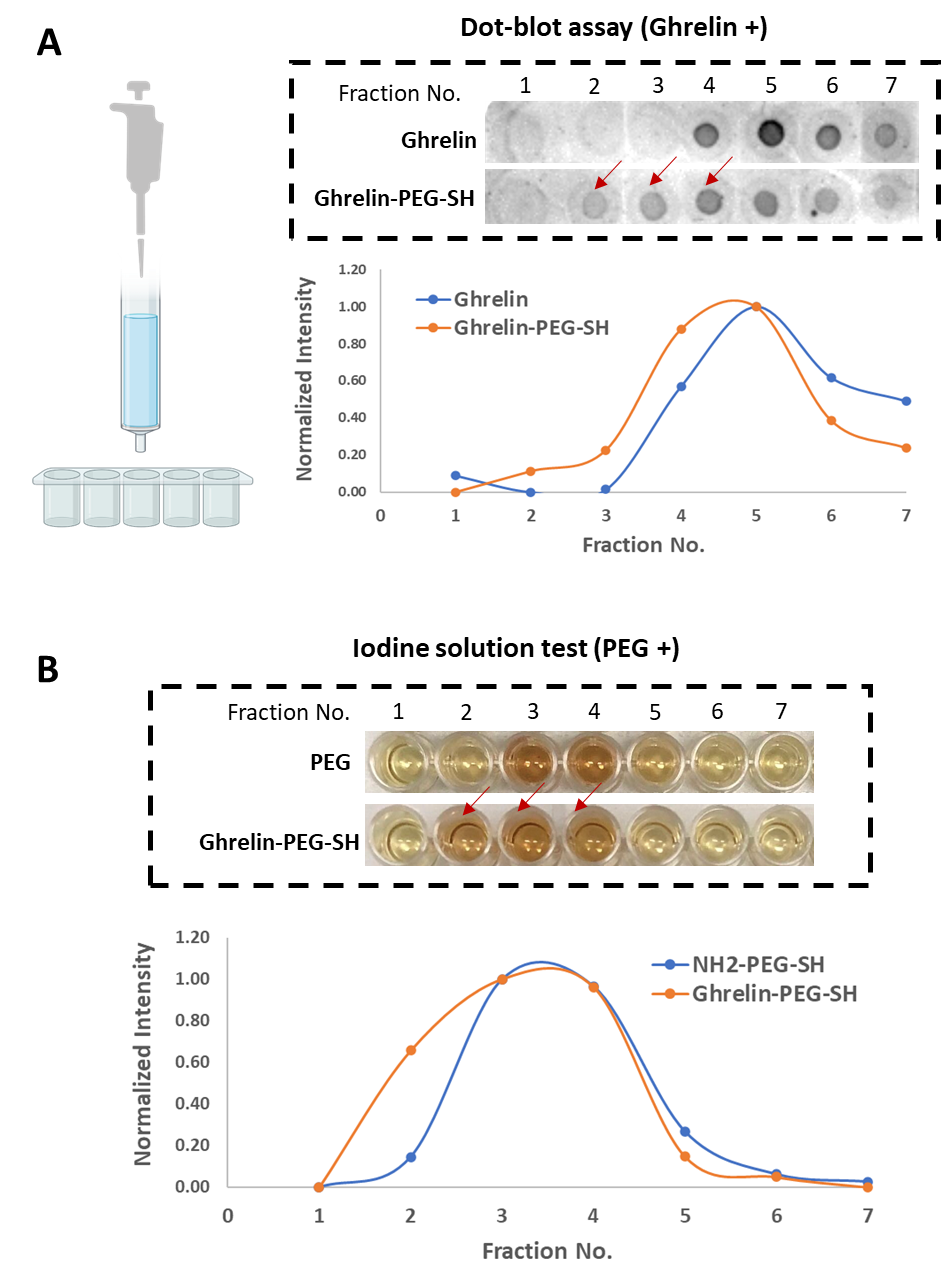
**

**Figure S2. Ghrelin-PEG-SH collection by size exclusion chromatography.** Ghrelin-PEG-SH synthesized at EDC: sulfo-NHS: ghrelin: NH_2_ -PEG-SH molar ratio of 20: 40: 1: 1 was loaded onto a NAP™-5 column packed with Sephadex G25 and eluted with deionized water. Factions containing 200 μL each were collected and analyzed by **(A)** dot blot immunostaining and **(B)** iodine solution straining to confirm the presence of ghrelin and PEG in the collected fractions. Ghrelin-PEG-SH eluted faster than ghrelin and NH_2_ -PEG-SH confirming the higher molecular weight of the conjugate. Ghrelin and NH_2_-PEG-SH were loaded as controls. Fractions 2-4 were collected as ghrelin-PEG-SH. Ghrelin-PEG-SH linkers were synthesized freshly each time before use.

**Table S1. Hydrodynamic size distribution of ghrelin-PEG-AuNRs.**

| **Compound** | **Mean size^[1]^** | **D10^[2]^** | **D50^[2]^** | **D90^[2]^** |
| --- | --- | --- | --- | --- |
|  | **(nm)** | | | |
| AuNRs | 46.1 ± 2.5 | 32.8 ± 0.6 | 39.3 ± 0.5 | 56.0 ± 3.5 |
| PEG-AuNRs | 79.2 ± 4.7 | 55.3 ± 0.7 | 68.3 ± 1.2 | 97.2 ± 2.4 |
| Ghrelin-PEG-AuNRs | 132.2 ± 9.5 | 74.1 ± 3.5 | 117.8 ± 2.8 | 191.3 ± 17.4 |

[1] Hydrodynamic size distribution was measured by nanoparticle tracking analysis (NTA).

[2] Percentile values. D10, D50 and D90 indicate the size below which 10%, 50% or 90% of all particles are found.


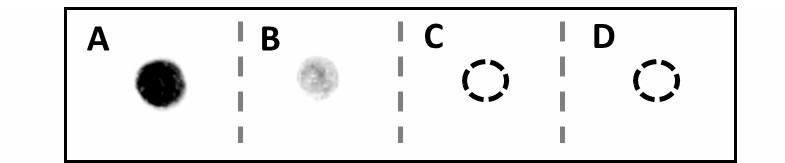


**Figure S3. Dot blot immunostaining for ghrelin presence analysis.** **(A)** Ghrelin-PEG-AuNRs, **(B)** Ghrelin and AuNRs reacted in the absence of NH_2_ -PEG-SH, **(C)** PEG-AuNRs and **(D)** AuNRs. Particles (4 µL, 10 nM of particles) after washing were spotted on NC membranes and gently dried under a nitrogen stream. The membranes were probed using primary ghrelin polyclonal antibody and HRP-conjugated secondary antibody. Only ghrelin-PEG-AuNRs stained positive for ghrelin by dot-blot immunostaining among ghrelin-PEG-AuNRs, PEG-AuNRs and AuNRs. Ghrelin alone showed little adsorption to AuNRs when a thiol functional group was absent in the structure.


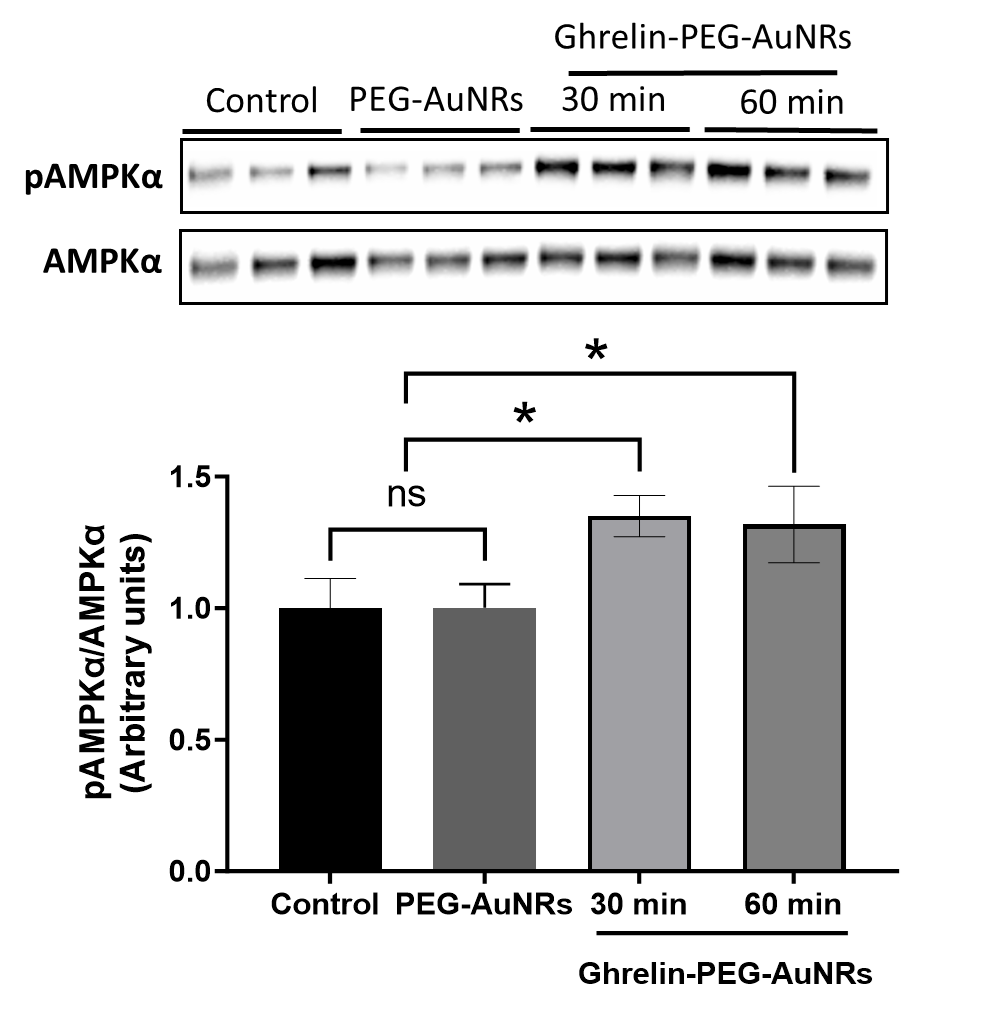


**Figure S4. Investigation of bioactivity of ghrelin-PEG-AuNRs following nose-to-brain delivery using Western blot analysis.** Ghrelin-PEG-AuNRs induced AMPK phosphorylation at 30 min and 60 min post-administration as evidenced by the representative Western blot images of pAMPKα and AMPKα and quantification of pAMPKα/AMPKα levels in brain lysates. The PEG-AuNR (no ghrelin) group was similar to the control group, suggesting AuNRs alone have no bioactivity in activation of AMPK phosphorylation. Values were expressed as mean ± SD, n=3. **P* < 0.05.

**Reference**

1. Gong, X.W., et al., *Discarded free PEG-based assay for obtaining the modification extent of pegylated proteins.* Talanta, 2007. **71**(1): p. 381-4.
